# Development of New Mouse Lung Tumor Models Expressing EGFR T790M Mutants Associated with Clinical Resistance to Kinase Inhibitors

**DOI:** 10.1371/journal.pone.0000810

**Published:** 2007-08-29

**Authors:** Lucia Regales, Marissa N. Balak, Yixuan Gong, Katerina Politi, Ayana Sawai, Carl Le, Jason A. Koutcher, David B. Solit, Neal Rosen, Maureen F. Zakowski, William Pao

**Affiliations:** 1 Pao Lab, Human Oncology and Pathogenesis Program, Memorial Sloan-Kettering Cancer Center, New York, New York, United States of America; 2 Varmus Lab, Cancer Biology and Genetics Program, Memorial Sloan-Kettering Cancer Center, New York, New York, United States of America; 3 Rosen Lab, Molecular Pharmacology and Chemistry Program, Memorial Sloan-Kettering Cancer Center, New York, New York, United States of America; 4 Imaging and Spectroscopic Physics Service, Department of Medical Physics, Memorial Sloan-Kettering Cancer Center, New York, New York, United States of America; 5 Solit Lab, Human Oncology and Pathogenesis Program, Memorial Sloan-Kettering Cancer Center, New York, New York, United States of America; 6 Department of Pathology, Memorial Sloan-Kettering Cancer Center, New York, New York, United States of America; 7 Department of Medicine, Weill Medical College of Cornell University, New York, New York, United States of America; Washington University, United States of America

## Abstract

**Background:**

The *EGFR T790M* mutation confers acquired resistance to kinase inhibitors in human EGFR mutant lung adenocarcinoma, is occasionally detected before treatment, and may confer genetic susceptibility to lung cancer.

**Methodology/Principal Findings:**

To study further its role in lung tumorigenesis, we developed mice with inducible expression in type II pneumocytes of *EGFR^T790M^* alone or together with a drug-sensitive *L858R* mutation. Both transgenic lines develop lung adenocarcinomas that require mutant EGFR for tumor maintenance but are resistant to an EGFR kinase inhibitor. *EGFR^L858R+T790M^*-driven tumors are transiently targeted by hsp90 inhibition. Notably, *EGFR^T790M^*-expressing animals develop tumors with longer latency than *EGFR^L858R+T790M^*-bearing mice and in the absence of additional kinase domain mutations.

**Conclusions/Significance:**

These new mouse models of mutant EGFR-dependent lung adenocarcinomas provide insight into clinical observations. The models should also be useful for developing improved therapies for patients with lung cancers harboring *EGFR^T790M^* alone or in conjunction with drug-sensitive *EGFR* kinase domain mutations.

## Introduction

Point mutations in the kinase domain of mutant epidermal growth factor receptors (EGFRs) are associated with acquired resistance to the EGFR inhibitors, gefitinib (Iressa) and erlotinib (Tarceva) in human lung adenocarcinoma [1]–[5]. The most common (>90%) second-site mutation involves a C→T change at nucleotide 2369 in exon 20, which results in substitution of methionine for threonine at position 790 (T790M). The amino acid change does not appear to diminish the catalytic activity of EGFR, but based upon crystal structure analyses, it is predicted to impair binding of either gefitinib or erlotinib to the EGFR ATP-binding pocket [6].

Although identified in the context of drug resistance, emerging data suggest that the T790M change may potentiate oncogenic activity, either by itself or in association with alterations in the EGFR kinase domain already known to confer gain-of-function properties [7]–[9]. Such alterations include deletions in exon 19 and point mutations in exon 21 (L858R). For example, although somatic *T790M* mutations in patients who never received gefitinib or erlotinib are rare [2], they can occasionally be found in tumors with primary drug resistance [10]. Second, rare cases of inherited susceptibility to lung cancer may be associated with a germline *T790M* mutation [11]. Third, we found the *T790M* mutation in an *EGFR^L858R^*-harboring lung adenocarcinoma cell line (H1975) that was developed and propagated in vitro prior to the development of EGFR kinase inhibitors [2]. Fourth, in a human bronchial epithelial cell line, overexpression of *EGFR^T790M^* confers a growth advantage over cells expressing wildtype *EGFR* [12]. Finally, the analogous substitution to T790M in BCR-ABL, T315I, is responsible for acquired resistance to ABL inhibitors in chronic myelogenous leukemia (CML) but has also been detected in CML patients before treatment [13].

To study further the role of the EGFR^T790M^ mutant in lung tumorigenesis in vivo, we have generated tetracycline (tet)-inducible transgenic mice that express in mouse lung epithelia the EGFR^T790M^ mutant alone or in conjunction with a TKI-sensitive EGFR^L858R ^mutant. We determined the effect of induction and de-induction of the transgenes in these animals by the exogenous administration and withdrawal, respectively, of the tet analog, doxycycline (dox). We further tested whether T790M-expressing lung tumors would respond to the kinase inhibitor, erlotinib, or an hsp90 inhibitor, 17-allylamino-17-demethoxygeldanamycin (17-AAG). The latter drug has been previously shown to selectively degrade mutant EGFR in cell culture and xenografts experiments [14], [15].

## Results

### Generation of mice with tet-regulatable *EGFR^L858R+T790M^* transgenes

A tet-inducible system has been used to regulate the expression in mouse lung epithelial cells of cDNAs encoding the commonly encountered mutant *EGFR* alleles, *EGFR^L858R^* and *EGFR^del L747-S752^*. Both mutants are associated with sensitivity to erlotinib [8], [9]. Here, to facilitate comparisons, we used the same tet-inducible system [9] to develop mouse lung tumor models that express the EGFR^T790M^ mutant.

**Figure 1:**
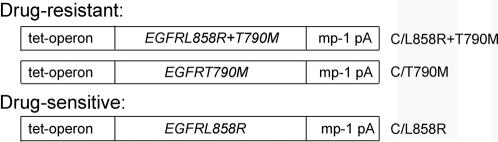
Design of transgenic constructs. Tet–tetracycline; mp-1 pA–poly A tract from the mouse protamine 1 gene; C–CCSP-rtTA. Bitransgenic mice harboring both *CCSP-rtTA* and *EGFR^L858R+T790M^* transgenes were labeled “C/L858R+T790M”. Bitransgenic mice harboring the *EGFR^T790M^* and *EGFR^L858R^* transgenes were labeled “C/T790M” and “C/L858R”, respectively. The latter strain of mice were previously described [9].

We first generated a double mutant *EGFR* allele encoding the *T790M* mutation associated with EGFR kinase inhibitor resistance together with the *L858R* mutation associated with drug sensitivity **(** **Figure 1** **)**. Transgene expression was induced in weaned double transgenic progeny (harboring the *CCSP-rtTA* and tet-regulated *EGFR^L858R+T790M^* transgenes; “C/L858R+T790M”) by administering dox via the animal diet [16]. Mice were subsequently screened at regular intervals via three ways: 1) for “clinical” signs possibly indicative of lung cancer (e.g. tachypnea and cachexia), 2) at the radiological level by magnetic resonance imaging (MRI) of mouse lungs, and/or 3) after sacrifice, at the histopathological level by analysis of lung sections. Among three founder lines identified with abnormal lung pathology (numbers 12, 29, and 51), one line (51) was particularly studied in further detail.

### Inducible, lung-specific expression of the mutant *EGFR* transgene in C/L858R+T790M mice

We observed that a bitransgenic mouse derived from line 51 became tachypneic and had an apparent large tumor burden on MRI after being administered a dox-containing diet for 17.5 weeks (data not shown). A colony from this line was subsequently expanded, and transgene-positive animals on dox for varying amounts of time were sacrificed for further analyses.

**Figure 2:**
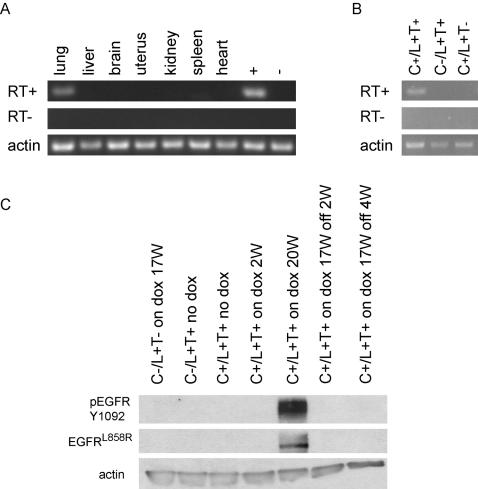
Inducible, lung-specific expression of the mutant *EGFR* transgene in C/L858R+T790M mice (line 51). A, B. RT-PCR performed in the presence or absence of reverse transcriptase (RT) using transgene-specific primers on mRNA from a bitransgenic animal on dox for 17.5 weeks (A) and various mice (genotypes as indicated) on dox for 5.5 weeks (B). “+” and “−“ denote known positive and negative lung samples derived from bitransgenic and non-transgenic mice on dox, respectively. “C/L+T” denotes C/L858R+T790M animals. C. Immunoblotting with antibodies against EGFR^L858R^, EGFR Y1092, and actin was performed on lung lysates derived from various mice on and/or off dox for varying periods of time; W–weeks. Genotypes are as indicated. “C/L+T” denotes C/L858R+T790M animals.

To determine whether mutant *EGFR* expression was specific to lung tissues from line 51 animals, we performed RT-PCR with transgene specific primers on mRNA extracted from various tissues derived from multiple progeny. Transgene expression was detectable only in lung tissue **(** **Figure 2A** **)**. Moreover, we could not detect the transgene in control mice, i.e. in animals that harbored only the *CCSP-rtTA* or *EGFR^L858R+T790M^* transgenes alone **(** **Figure 2B** **)**.

Immunoblotting studies with an anti-EGFR^L858R^-specific antibody [9] were next performed on extracts of lungs from various non-, mono-, or bi-transgenic animals fed either a normal or dox-containing diet. While mutant EGFR protein was detected in lungs from C/L858R+T790M mice fed dox for 20 weeks, none was detected in lungs from control animals **(** **Figure 2C** **)**. Mutant EGFR protein was also not detected in lung lysates from mice on dox for 17 weeks (with documented radiographic lung lesions; see below) and then subsequently fed a normal diet for 2 and 4 weeks, respectively. Expression of the mutant protein correlated with the detection of EGFR autophosphorylation at an important tyrosine residue (Y1092) **(** **Figure 2C** **)**. The timepoint at which induction of EGFR^L858R+T790M^ protein can first be detected after initiation of dox appears to be somewhat variable, because we were able to detect the mutant protein after 2 weeks of induction in lungs from some animals (data not shown) but not in others **(** **Figure 2C** **)**. RT-PCR performed on lung-derived mRNA showed similar results regarding transcription of the transgene (data not shown). Nevertheless, these results collectively indicate that we were able to achieve inducible, lung-specific expression of mutant EGFR in C/L858R+T790M mice.

### C/L858R+T790M mice on dox develop lung adenocarcinomas

**Figure 3:**
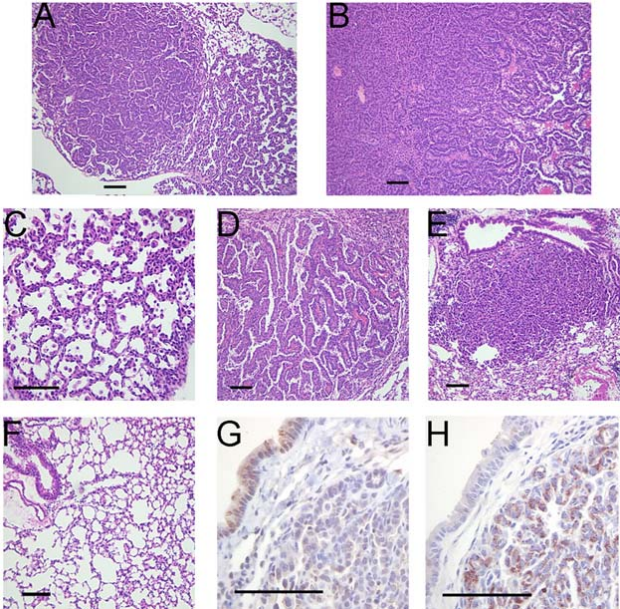
C/L858R+T790M mice fed doxycycline develop lung adenocarcinomas. Hematoxylin and eosin (H&E)-stained sections (A–F) of lungs derived from three different bitransgenic progeny of line 51 displaying different adenocarcinoma histological subtypes, including solid (A), bronchioloalveolar (A, C), and papillary (B, D), and one of line 12 with solid and bronchioloalveolar subtypes (E), fed dox for 11 (A, C), 16 (D), 17.5 (B), and 32 weeks (E), respectively. Lungs from bitransgenic mice fed a normal diet are shown in F. Lung tumors are composed mainly of type II pneumocytes, as indicated by the lack of staining for the Clara cell marker, CC26 (G) and positive staining for the type II cell marker, surfactant protein-C (SP-C) (H). Bars, 100 microns.

We analyzed multiple untreated bitransgenic mice from line 51 fed dox for varying amounts of time (0–32 weeks), to determine the effect of mutant EGFR protein expression on mouse lungs **(** **Figure 3** **)**. Lungs from bitransgenic mice maintained without dox exhibited normal lung morphology **(** **Figure 3F** **)**. By contrast, lungs from mice fed dox displayed heterogeneous adenocarcinomas **(** **Figures 3A–D** **)** involving three main histological subtypes: solid **(** **Figure 3A** **)**, bronchioloalveolar **(** **Figures 3A, C** **)**, and papillary **(** **Figures 3B, D** **)**. Often, multiple histological subtypes were observed in the same mouse in adjacent lesions **(** **Figures 3A, B** **)**. Immunohistochemical analyses revealed that tumors were negative for CC26, a Clara cell protein **(** **Figure 3G** **)** and positive for surfactant protein-C, a type II pneumocyte marker **(** **Figure 3H** **)**, indicating that the tumor cells had a type II cell-like phenotype. Tumors were not examined for the presence of bronchioalveolar stem cells, which display features of both type II pneumocytes and Clara cells [**17**].

**Figure 4:**
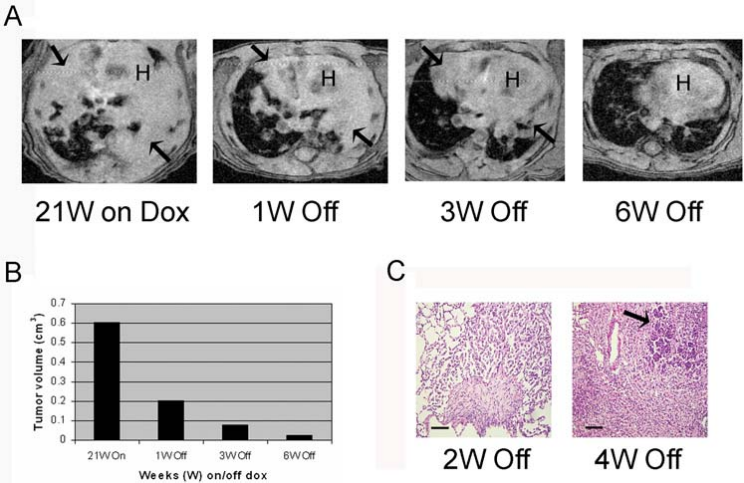
Lung tumors in C/L858R+T790M mice require expression of mutant EGFR for tumor maintenance. A. Serial axial lung field MR images from a mouse administered dox for 21 weeks (W) and then fed a normal diet for the indicated amounts of time. Arrows indicate lung opacities; H, heart. B. Tumor volumes were quantified in the images depicted in A, using imaging software. See methods for details. C. H&E-stained sections from lungs of bitransgenic mice on dox for 17 weeks with tumor by MRI and then taken off dox for the indicated times. Left–degenerating tumor surrounding a scar; right–dying tumor cells surrounded by an inflammatory response; arrowed–island of viable tumor cells.

We previously used MRI to detect and monitor lung lesions in other lung tumor models [9], [18]. In EGFR^L858R+T790M ^animals, lung lesions appeared mostly as dense solid consolidations **(** **Figures 4A** **and** **5A** **)**. The patterns observed by MRI correlated well with tumor histopathology (data not shown).

**Figure 5:**
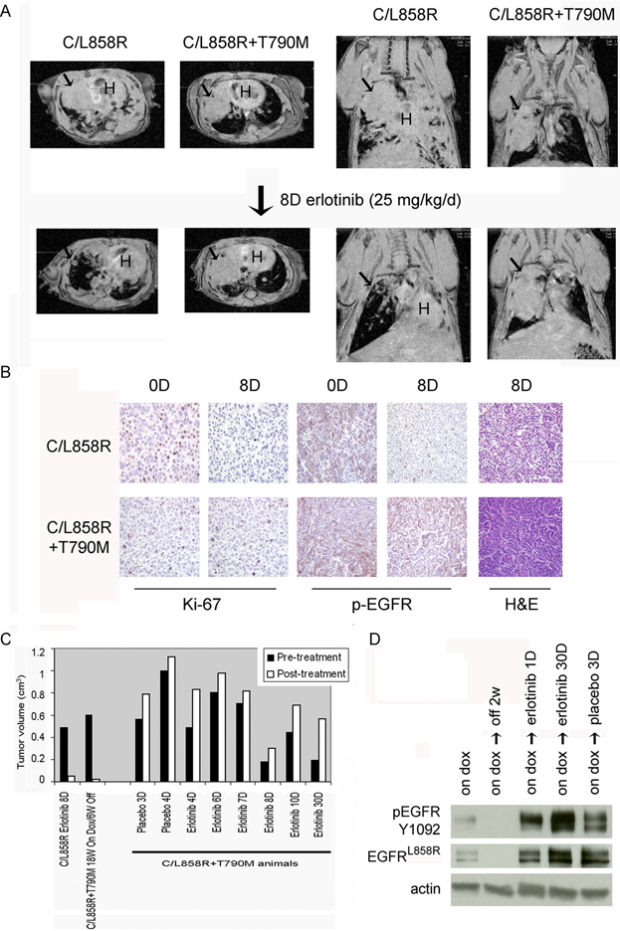
Lung tumors in C/L858R+T790M mice are resistant to treatment with erlotinib. A. Serial axial (left) and coronal (right) images from C/L858R or C/L858R+T790M mice, treated with erlotinib for 8 days. Arrow depicts tumor; H–heart. B. Histological sections derived from the lungs of C/L858R (upper panel) or C/L858R+T790M (lower panel) mice treated with erlotinib for 0 or 8 days. Tumors were stained for the proliferation marker, Ki-67, and phospho-EGFR (pEGFR, Y1016). Far right: H&E staining reveals significant treatment effect in a representative C/L858R tumor, but viable cells in the corresponding C/L858R+T790M tumor. C. Tumor volumes were quantified in the MR images obtained from the individual mice pre- and post-treatment with placebo or erlotinib. See Table S1 and methods for details. For comparison, tumor volumes from a drug-sensitive C/L858R mouse pre- and post-erlotinib treatment and from a C/L858R+T790M mouse on and off dox for the indicated times are also displayed. W–weeks; D–days. D. Immunoblotting with antibodies against EGFR^L858R^, EGFR Y1092, and actin was performed on lysates derived from lungs of tumor-bearing C/L858R+T790M mice, treated with placebo or erlotinib for the indicated times.

### Expression of mutant EGFR is required for tumor maintenance in C/L858R+T790M animals

To examine the dependence of EGFR^L858R+T790M^-induced lung tumors on continued expression of the mutant *EGFR* transgene, we monitored lung lesions by MRI in six bitransgenic animals (on dox for 13–32 weeks) after dox was removed. Serial MRIs showed that lung tumors progressively regressed in all animals **(** **Figure 4A** **and data not shown)**. Regression was seen as early as one week after removal of the inducer. By six weeks, MRI lesions appeared to have mostly resolved **(** **Figure 4A** **)**. The serial change over time in lung opacities seen by MRI–presumably indicative of tumor volume (cm^3^) in an animal -- was readily quantifiable in individual animals using imaging software (see methods and **Figure 4B** **)**.

In animals sacrificed after dox withdrawal, lungs displayed mostly degenerating tumors at the histological level **(** **Figure 4C** **)**. Consistent with this observation, immunoblotting of lung lysates from the corresponding animals showed no detectable expression of mutant EGFR **(** **Figure 2C** **)**. However, microscopic islands of tumor cells could still be detected on tissue sections, even after mice were off dox for four weeks **(** **Figure 4C, right panel** **)**.

### Lung tumors in C/L858R+T790M animals are resistant to treatment with erlotinib

We next examined the effect of erlotinib on lung tumors in C/L858R+T790M animals. Tumor-bearing animals with large lung opacities on MRI were administered placebo (n = 3) or erlotinib (n = 8) **(Table S1 and** **Figure 5** **)**. The EGFR inhibitor was given for 1 to 30 days at doses (25–50 mg/kg/d) known to be effective at inducing regressions in C/L858R mice that bear drug-sensitive tumors [9]. During the treatment period, many of the animals became progressively dyspneic and had to be sacrificed at timepoints earlier than originally planned. Among six evaluable C/L858R+T790M animals assessed radiographically, none responded to erlotinib **(Table S1 and** **Figure 5C** **)**.

The radiological data were further supported by subsequent histological analyses of tumors from treated mice. While drug-sensitive L858R-bearing lungs showed degenerating tumor after erlotinib [9] **(** **Figure 5B** **)**, EGFR^L858R+T790M^-bearing tumors contained virtually all viable cells with no treatment effect **(** **Figure 5B** **and Table S1).** In addition, while the L858R-bearing tumors displayed a greater than 10-fold decrease in proliferation, the EGFR^L858R+T790M^-bearing tumors did not **(** **Figure 5B** **and Figure S1)**. Furthermore, while L858R-treated tumor cells demonstrated a dramatic decrease in phospho-EGFR staining **(** **Figure 5B** **)**, the treated C/L858R+T790M tumor cells continued to display reactivity with phospho-EGFR antibody. Consistent with this, immunoblotting analyses indicated that lysates made from lungs of drug-treated C/L858R+T790M animals continued to display reactivity with phospho-EGFR-specific antibodies **(** **Figure 5D** **)**. In total, none of the eight erlotinib-treated animals displayed evidence of significant response to the kinase inhibitor. When compared with drug-sensitive C/L858R mice, where 12 of 12 mice displayed tumor responses at doses ≥12.5 mg/kg/day [9], the difference in response rates (100% vs 0%) was statistically significant (p<0.0001, Fisher's exact test).

To extend our findings to a separate line of C/L858R+T790M animals, we treated a lung adenocarcinoma-bearing mouse **(**e.g. **Figure 3E** **)** derived from a different founder (#12) with erlotinib at 50 mg/kg/d for 12 days. Consistent with the results discussed above, lung tumors in this animal did not show a response at either the radiological or histological levels (data not shown).

### Lung tumorigenesis in C/T790M mice

Using the same transgenic plasmid construct **(** **Figure 1** **)** and bitransgenic system as described above, we also generated mice expressing tetracycline-regulatable alleles carrying a mutant *EGFR^T790M^* cDNA alone. Thus far, we have identified 5 promising founder lines (8, 22, 37, 45, and 71); lines 8 and 37 have been characterized in most detail.

**Figure 6:**
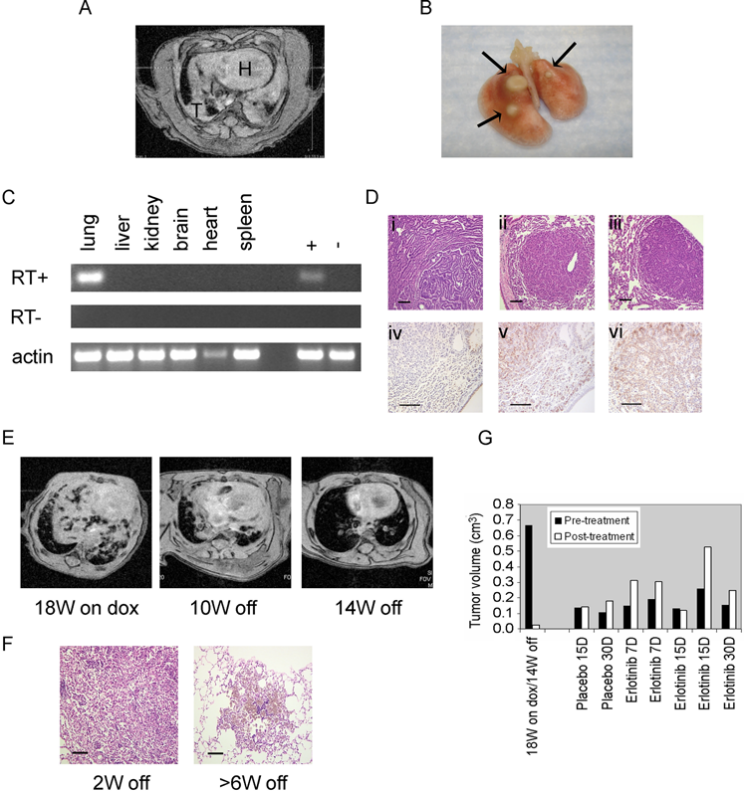
Characteristics of C/T790M mice. A. Axial MR image from a line 37-derived bitransgenic animal fed dox for 32 weeks, revealing tumor (T); H: heart. B. Gross histology shows corresponding lung lesions. C. RT-PCR was performed on lungs from this animal in the presence or absence of RT using transgene-specific primers on mRNA from various tissues; “+”–positive control; “−“– negative control. D. Images of H&E-stained lung sections from various T790M transgene bearing mice. Lungs from bitransgenic line 37 (i) and 8 animals (ii-iii) displayed features of papillary adenocarcinoma (i), solid adenocarcinoma surrounded by bronchioloalveolar carcinoma (ii), and solid/papillary adenocarcinoma surrounded by bronchioloalveolar carcinoma (iii). Tumors were negative for CC26 (iv), positive for SP-C (v), and positive for phospho-EGFR (Y1016) (vi). Bars, 100 microns. E. Serial MR images of a bitransgenic C/T790M animal (line 8) administered dox 18 weeks (left) and then fed a normal diet for the indicated times; W–weeks. F. H&E-stained sections from lungs of bitransgenic tumor-bearing mice withdrawn from dox for the indicated times. Left–degenerating tumor with scattered viable cells; Right–hemosiderin-laden macrophages engulfing a degenerating tumor. G. Tumor volumes were quantitated in the MR images obtained from the individual mice pre- and post-treatment with placebo or erlotinib. See Table S2 and methods for details. W–weeks, D–days.

Bitransgenic progeny on dox were screened for lung tumors as above. In most instances, animals from these lines developed detectable lesions only after long-term administration of dox, and we never observed the development of tachypnea or cachexia. In mice derived from line 37, lung lesions were not visible by MRI until about 28–32 weeks on dox **(** **Figure 6A** **)**. These lesions corresponded well with gross lung histology **(** **Figure 6B** **)**. In line 8, while one animal was found to have visible lung lesions after 18 weeks on dox, another 5 animals developed lung lesions only after 28–32 weeks. In lines 22, 45 and 71, lesions were detected after 52, 40 and 32 weeks, respectively (data not shown).

Similar to C/L858R+T790M animals, C/T790M animals (lines 37 and 8) displayed lung-specific expression of the mutant *EGFR* transgene **(** **Figure 6C** and data not shown**)**. H&E staining of lung sections revealed invasive lung adenocarcinomas with a histological spectrum similar to those in C/L858R+T790M mice **(** **Figure 6D** **)**. Moreover, C/T790M tumors were dependent upon mutant EGFR for survival, as tumors regressed after dox withdrawal **(** **Figures 6E and 6F** **)**. Finally, as expected, tumors did not respond to erlotinib; none of five animals treated for 7 to 30 days with 50 mg/kg/d showed any tumor response **(Table S2 and** **Figure 6G** **)**.

Others have reported that in a family with multiple cases of lung adenocarcinoma associated with germline transmission of the *T790M* mutation, four of six tumors analyzed by dideoxynucleotide sequencing showed a secondary somatic activating EGFR mutation, arising in cis with the germline *T790M* mutation [11]. To determine if tumors that arose in C/T790M animals similarly had a secondary somatic activating EGFR mutation on the same allele as the *T790M* mutation, we used oligonucleotide primers that span the human *EGFR* kinase domain to perform transgene-specific RT-PCR on mRNA derived from nine lung tumor nodules derived from nine different animals (3 from line 37, 5 from line 8, and 1 from line 22). Sequence analysis of the PCR products revealed the presence of the *T790M* mutation, but no additional kinase domain mutations were observed. Although we cannot currently exclude the possibility of other cooperating oncogenes that contribute to the lung tumors in these animals, these data indicate that expression of *EGFR^T790M^* alleles in mouse lung can lead to lung tumor formation in vivo in the absence of more common gain-of-function EGFR kinase domain mutations (i.e. deletion mutations in exon 19 or the common *L858R* point mutation in exon 21).

**Figure 7:**
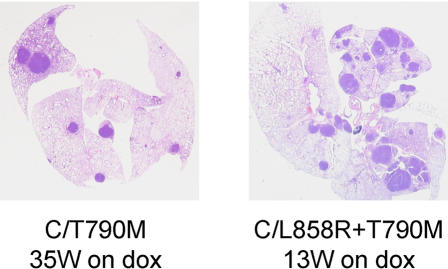
Comparison of phenotypes induced by various EGFR mutants. Representative pictures of H&E-stained lung sections from C/T790M (line 8) and C/L858R+T790M (line 51) animals, fed dox for the indicated amounts of time. W–weeks.

Interestingly, C/L858R+T790M (line 51) and C/T790M (line 8) animals on dox developed lung tumors with different latencies (∼17 vs ∼32 weeks). Moreover, despite being on dox for less time, the number of nodules per lung that developed in C/L858R+T790M animals was greater than those observed in C/T790M mice **(** **Figure 7** **)**. These data are consistent with the notion that the T790M mutation, when combined with activating EGFR kinase domain mutations, confers enhanced catalytic phosphorylating activity, as shown by others using insect cells infected with baculovirus expressing various EGFR intracellular domain constructs [19].

### Antitumor activity induced by the hsp90 inhibitor, 17-AAG, in C/L858R+T790M mice

One goal of developing mouse tumor models that express EGFR^T790M^-driven lung adenocarcinomas is to use them to identify agents that can potentially overcome T790M-mediated resistance. Thus, as proof-of-principle, we treated tumor-bearing C/L858R+T790M animals with the geldanamycin analogue, 17-AAG. This ansamycin antiobiotic acts by inhibiting hsp90, a molecular chaperone which helps the folding of nascent polypeptides and stabilizes oncogenic kinases [20]. It has been shown in cell culture and xenograft models that 17-AAG can degrade mutant EGFRs, including EGFR^L858R+T790M^ [14], [15].

**Figure 8:**
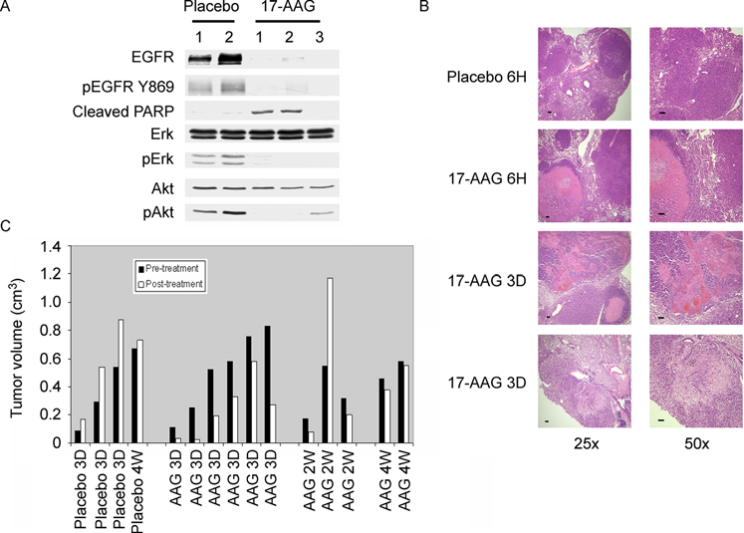
Effect of 17-AAG on lung tumors from C/L858R+T790M animals. A. Immunoblotting analyses of lung lysates derived from bitransgenic animals (line 51) treated with placebo or 17-AAG and sacrificed 6 hours later. Lane numbers correspond with the animal study numbers in Table S3. Lysates were probed with the indicated antibodies; p-phospho. B. Histology of lung tumors from bitransgenic mice treated with either placebo or 17-AAG for indicated times; H–hours; D–days. Lungs from placebo treated mice had viable tumor (upper panels), while lungs from drug-treated animals displayed areas of tumor necrosis surrounded by rims of viable cells (two middle panels), and degenerating tumors with inflammatory response (lower panels). Bars, 100 microns. C. Tumor volumes were quantitated in the MR images obtained from the individual mice pre- and post-treatment with placebo or 17-AAG. See Table S3 and methods for details. D–days; W–weeks.

We first treated tumor-bearing C/L858R+T790M animals with either placebo or 17-AAG, sacrificed mice 6 hours later, and examined the levels of total EGFR in lung lysates. As compared to extracts from lungs of mice treated with placebo, levels of EGFR were lower in extracts from lungs of drug-treated mice **(** **Figure 8A** **)**, consistent with the notion that 17-AAG induces degradation of EGFR. Degradation was accompanied by variable effects on the phosphorylation of downstream components of the EGFR signaling pathway (i.e. Erk and Akt) **(** **Figure 8A** **)**. At the histological level, lungs from 6h-treated animals displayed evidence of treatment effect such as tumor necrosis **(** **Figure 8B** **)**. In contrast, lungs from placebo-treated animals displayed only viable tumor **(** **Figure 8B** **)**.

To assess the durability of 17-AAG-induced treatment, additional animals were treated with 75 mg/kg/day of drug for 3 consecutive days per week for 1 week (n = 6), 2 weeks (n = 3), or 4 weeks (n = 2). Control animals were treated with placebo for 3 consecutive days per week for 1 week (n = 3) or 4 weeks (n = 1). This dosing schedule was previously shown to deliver the maximum tolerated dose to mice [21]. Mice were assessed for responses by MRI. Whereas 3 of 3 animals treated with placebo displayed progressive disease, five of six animals treated with drug for 3 days displayed partial responses **(Table S3,** **Figure 8C** **and Figure S2)**. Corresponding lung tumor tissue sections from responding animals displayed tumor necrosis **(** **Figure 8B** **)**. However, in most treated animals, viable tumor nodules remained **(** **Figure 8B** and data not shown). After 2 weeks of treatment, two of three animals displayed partial responses, and after 4 weeks of treatment, two of two animals displayed stable disease **(Table S3,** **Figure 8C** **and Figure S2)**. A transient antitumor effect was also observed in tumor-bearing C/L858R mice (that express the erlotinib-sensitive EGFR mutant) that were similarly treated with 17-AAG (data not shown). Collectively, these data indicate that the hsp90 inhibitor, 17-AAG, has some antitumor effect against T790M-driven lung tumors. However, the dosing schedule used for these experiments induced only unsustained and modest disease control.

## Discussion

Mutations that lead to substitution of methionine for threonine at position 790 in the EGFR kinase domain have been found in about 50% of tumors from patients with acquired resistance to the EGFR inhibitors, gefitinib and erlotinib [1]–[5]. *EGFR^T790M^* alleles also have been detected in a minority of tumors with primary resistance to these drugs [10]. Although such mutations are predominantly somatic, a family with a germline *T790M* mutation has also been described [11]. These human genetic data underscore the significance of this particular mutation in human lung cancer, especially in relation to targeted therapies.

In order to understand further the role of the *EGFR^T790M^* mutation in lung tumorigenesis, we have developed two new inducible mouse models of lung cancer that express transgenes encoding either *EGFR^T790M^* or *EGFR^L858R+T790M^*. The latter construct encodes the T790M mutant in combination with the drug-sensitive L858R mutant. Expression of either transgene induces formation of heterogenous lung adenocarcinomas that display histologies commonly found in human NSCLC, i.e. solid, papillary, and bronchioloalveolar subtypes. Tumors from mice expressing either allele are dependent upon mutant EGFR for survival. Moreover, compared to mice that express the drug-sensitive alleles, *EGFR^L858R^* and *EGFR^del L747-S752^* [9], *EGFR^T790M^-* and *EGFR^L858R+T790M^*-driven tumors are resistant to erlotinib.

Mice expressing the *EGFR^T790M^* transgene alone usually develop tumors with longer latency than animals expressing *EGFR^L858R+T790M^* (shown here) or even *EGFR^L858R^* alone [9]. Importantly, tumor nodules from multiple C/T790M mice from different founder lines do not appear to harbor secondary kinase domain mutations arising in cis. These findings collectively demonstrate that the mutant EGFR^T790M^ can induce lung tumor formation in the absence of additional gain-of-function EGFR kinase domain mutations. However, because C/T790M mice develop tumors with a relatively long latency, it is likely that other genes may collaborate with *EGFR^T790M^* to induce lung tumorigenesis.

How EGFR^T790M^ facilitates transformation remains to be fully determined. Studies using global phosphoproteome analysis of kinase inhibitor-resistant BCR-ABL mutants suggest that the analogous T315I mutation in BCR-ABL substantially alters kinase function and is associated with a unique phosphosubstrate signature, such as a shift in phosphorylation of two tyrosines in the P-loop of BCR-ABL [22]. It will be interesting in the future to determine if EGFR^T790M^ similarly is associated with a unique phosphosubstrate signature in EGFR. Such studies could provide insight into disease progression as well as uncover new targets for therapy.

Finally, one aim of developing mouse tumor models that express EGFR^T790M^-driven lung adenocarcinomas is to use them to identify agents that can potentially overcome T790M-mediated resistance. As proof-of-principle, we treated mice with the hsp90 inhibitor, 17-AAG, which can target mutant EGFRs, at least in cell culture or xenograft studies [14], [15]. Our results demonstrate that 17-AAG does have some modest antitumor activity against EGFR^L858R+T790M^-dependent tumors, as manifested by EGFR degradation, in vivo radiographic responses, and histological evidence for treatment effect. However, most tumor nodules in mice remained viable, and any responses seen by MRI appeared to be transient. Such results could be due to the short half-life of 17-AAG (6 hours) and an inability to dose the drug more frequently due to hepatotoxicity [21]. However, these data are consistent with xenograft model systems, where, compared to placebo, 17-AAG treatment alone delayed but did not inhibit the growth of xenografts comprised of H1975 cells (*p* value >0.05; Sawai, Solit, and Rosen, unpublished). These cells harbor *EGFR^L858R+T790M^* and are resistant to gefitinib/erlotinib in vitro [2]. In the future, we hope to find agents that can induce sustained tumor responses in these lung tumor-bearing animals.

T790M-mediated resistance to EGFR inhibitors remains a significant clinical problem. We have generated two new mouse models that provide insights into clinical observations. The models could help accelerate the development of improved therapies for patients with lung cancers harboring *EGFR^T790M^* alone or in conjunction with drug-sensitive *EGFR* kinase domain mutations.

## Materials and Methods

### Generation of transgenic mice

*EGFR* cDNAs encoding *EGFR^T790M^* or *EGFR^L858R+T790M^* [2] were excised from pcDNA3.1(-) with *Pme*I and ligated into the *tet-op-mp1* vector [18] via the EcoRV site, downstream of a tetracycline-responsive element. To ensure stability of the mRNA, the 3′ end of the construct contains an intron and polyadenylation sequence derived from the mouse protamine-1 gene. An extraneous 100 bp SalI fragment, between the tet element and the ATG of *EGFR*, was further excised from each construct as previously described [9]. Entire DNA transgenes were released using *Bss*HII and injected into fertilized FVB F2 eggs by the MSKCC Transgenic Core Facility. For the *EGFR^L858R+T790M^* transgene, 60 pups were obtained. For the *EGFR^T790M^* transgene, two separate egg injections produced 212 pups. Tail PCR genotyping and/or Southern blotting identified six and twenty-four founders, respectively, for each transgene. Founders were subsequently crossed to mice that express the reverse tetracycline transactivator (rtTA) in type II pneumocytes (i.e. CCSP-rtTA) [23]. The latter animals were also on an FVB background.

### Animal husbandry and genotyping

All animals were housed in specific pathogen-free housing with abundant food and water, and they were treated with various drugs under guidelines approved by the MSKCC Institutional Animal Care and Use Committee and Research Animal Resource Center. *CCSP-rtTA* mice were previously described [23]. Doxycycline was administered by feeding mice with dox-impregnanted food pellets (625 ppm; Harlan-Teklad). TetO-EGFR transgenic mice were genotyped using tail DNA, isolated with the Qiaprep Tail DNeasy isolation kit (Qiagen), with the following primers: EGFR3421, 5′-ACTGTCCAGCCCACCTGTGT-3′; and mp-1R, 5′-GCCTGCGACGGCGGCATCTGC-3′. Reactions were amplified with the following PCR protocol: denaturation for 5 min at 95°C, followed by 35 cycles of 30 sec at 95°C, 30 sec at 58°C, and 30 sec at 72°C, followed by a 5-min extension at 72°C. Transgenic founders were further confirmed by Southern blotting of tail DNA, using a ∼500 bp XhoI-SalI probe at the 5′ end of the construct.

Erlotinib (kindly provided by Genentech) was suspended in 0.5% (w/v) methylcellulose and injected intraperitoneally at the doses and times indicated. 17-AAG (kindly provided by Conforma Therapeutics, now Biogen Idec, Cambridge MA) was dissolved in DMSO to yield 50mg/mL stock solutions and stored at −20°C. The EPL diluent was obtained from the Drug Synthesis and Chemistry Branch, Developmental Therapeutics Program, National Cancer Institute. Prior to injection, 17-AAG was diluted with EPL solution at a minimum of 1:3 and administered by i.p. injection.

### Histology

Animals were sacrificed with a lethal dose of CO_2_ per institutional guidelines. In most instances after excision, the left lung was flash-frozen in liquid nitrogen. The right lung was inflated with 4% paraformaldehyde in PBS, fixed overnight at room temperature, placed in 70% ethanol, and sent for paraffin embedding and sectioning (Histoserv, Gaithersburg, MD). In some animals, gross tumor nodules were macrodissected and flash-frozen, and the remaining lung tissues were processed for histological analysis. All lungs were sectioned in the same manner: 5 steps were taken, 100 microns apart. All steps were evaluated to determine whether tumors were present. Slides were reviewed by a board-certified pathologist with expertise in lung cancer (MFZ).

### RT-PCR analysis

RNA was extracted from pulverized tissue samples using Trizol (Invitrogen) reagent. RNA was treated with DNase I (Sigma) to eliminate contaminating DNA. RT-PCR reactions were performed using the SuperScript III One-Step RT-PCR with Platinum *Taq* system (Invitrogen), with the following primers: RT-PCR F: 5′-ACCAAGCCACAGCAGGTCCT-3′ and RT-PCR R 5′-TATGGTGTATGAGCGGCGGC-3′. Control reactions were performed with Platinum *Taq* polymerase in the absence of reverse transcriptase.

### Mutational analysis

To seek potential mutations in the coding sequence of the *EGFR* kinase domain, RT-PCR analysis was performed as described above on mRNA from discrete lung nodules macrodissected from various mice. The following primers that span exons 18–21 were used: 2101F: 5′-CCCAACCAAGCTCTCTTGAG-3′ and 2948R: 5′-AATGACAAGGTAGCGCTGGGGG-3′. PCR products were then analyzed by direct dideoxynucleotide sequencing. All sequence tracings were manually reviewed in the forward and reverse directions.

### Antibodies and immunoblotting

For experiments involving 17-AAG, tumor lysates were homogenized in SDS lysis buffer (50mM Tris-HCl (pH7.4) and 2% SDS). For all other immunoblotting experiments, established protocols were performed [24]. The following antibodies against specific proteins were used: EGFR, phospho-EGFR (Y869, Y1016, 1092), Akt, phospho-Akt, MAPK, phospho-MAPK (Cell Signaling, Beverly, CA); and total EGFR (sc-03; Santa Cruz). The L858R-antibody was previously described [9]. Note: two numbering systems exist for EGFR. The first denotes the initiating methionine in the signal sequence as amino acid -24. The second, used here, denotes the methionine as amino acid +1.

### MRI

Mice were imaged according to established protocols by the MSKCC Small Animal Imaging Core facility [9]. Briefly, animals were anesthetized with 2% isoflurane oxygen gas. Two dimensional (axial and coronal) MRI was then performed on a Bruker 4.7T Biospec scanner (Bruker Biospin Inc.), using a custom-made 36mm quadrature birdcage coil. “Tumor volume” (cm^3^) per animal was quantified by calculating the area of visible lung opacities present in each axial image sequence (usually 20–22 per mouse), using ParaVision 3.0.2 imaging software, and then multiplying the total sum of the areas by 0.09 cm (the distance between each MRI sequence).

There are no standard response criteria for evaluating the effect of drug treatment on lung tumors in mice. In humans, such criteria are based on uni- or bidimensional measurements, obtained from imaging studies where patients are conscious, placed in a certain position, and asked to hold their breaths at specific times. In mice, which are anesthesized, we have found that uni- or bi-dimensional measurements are not as accurate to assess tumor responses (data not shown). Therefore, we used tumor volume measurements and the following criteria to classify tumor responses to treatment: 1) complete response (CR): the disappearance of all target lesions; 2) partial response (PR): at least a 30% decrease in the volume of target lesions, taking as reference the baseline tumor volume; 3) progressive disease (PD): at least a 20% increase in the volume of target lesions, taking as reference the baseline tumor volume, and 4) stable disease (SD): neither sufficient shrinkage to qualify for partial response nor sufficient increase to qualify for progressive disease, taking as reference the baseline tumor volume.

## Supporting Information

Table S1 Summary of C/L858R+T790M bitransgenic mice (line 51) treated with erlotinib.
(0.06 MB DOC)

Table S2 Summary of C/T790M bitransgenic mice treated with erlotinib.
(0.05 MB DOC)

Table S3 Summary of C/L858R+T790M bitransgenic mice (line 51) treated with 17-AAG.
(0.09 MB DOC)

Figure S1 Status of cell proliferation in lung tumors from C/L858R and C/L858R+T790M animals treated with erlotinib. Histological sections derived from the lungs of bitransgenic animals were stained with antibodies to the proliferation marker, Ki-67. Ki-67-positive cells in lungs were then quantitated by determining the average (with standard deviations) number of positive cells counted in three separate high power fields (hpf) at a magnification of 200x.
(3.34 MB TIF)

Figure S2 Representative MR images from bitransgenic mice treated with 17-AAG. Serial pre- and post-treatment images are shown. D-days; W-weeks.
(7.55 MB TIF)
