# Supplementary material for: Development of New Mouse Lung Tumor Models Expressing EGFR T790M Mutants Associated with Clinical Resistance to Kinase Inhibitors
Source: PLoS One. 2007 Aug 29;2(8):e810. doi: 10.1371/journal.pone.0000810 (PMC1950079; doi:10.1371/journal.pone.0000810)
Supplement: Table S1 — Summary of C/L858R+T790M bitransgenic mice (line 51) treated with erlotinib. (0.06 MB DOC) [file pone.0000810.s001.doc]

| **Mouse** | **Erlotinib dose**  **(mg/kg/d)** | **Duration of treatment (days)** | **Response by MRI** | **Histology after erlotinib treatment** |
| --- | --- | --- | --- | --- |
| 1 | Placebo | 1 | nd | Viable tumor |
| 2 | Placebo | 3 | PD | Viable tumor |
| 3 | Placebo | 4 | SD | Viable tumor |
|  | | | | |
| 1 | 25 | 3 | nd | Viable tumor |
| 2 | 25 | 8 | PD | Viable tumor |
| 3 | 50 | 1 | nd | Viable tumor |
| 4 | 50 | 4 | PD | Viable tumor |
| 5 | 50 | 6 | PD | nd |
| 6 | 50 | 7 | SD | Viable tumor |
| 7 | 50 | 10 | PD | nd |
| 8 | 50 | 30 | PD | Viable tumor |

**Table S1. Summary of C/L858R+T790M bitransgenic mice (line 51) treated with erlotinib.** nd – not determined; PD – progressive disease; SD – stable disease. Responses were defined as described in the Methods.
