# Supplementary material for: Development of New Mouse Lung Tumor Models Expressing EGFR T790M Mutants Associated with Clinical Resistance to Kinase Inhibitors
Source: PLoS One. 2007 Aug 29;2(8):e810. doi: 10.1371/journal.pone.0000810 (PMC1950079; doi:10.1371/journal.pone.0000810)
Supplement: Table S2 — Summary of C/T790M bitransgenic mice treated with erlotinib. (0.05 MB DOC) [file pone.0000810.s002.doc]

| **Mouse** | **Line** | **Erlotinib dose**  **(mg/kg/d)** | **Duration of treatment (days)** | **Response by MRI** | **Histology after erlotinib treatment** |
| --- | --- | --- | --- | --- | --- |
| 1 | 8 | Placebo | 15 | SD | Viable tumor |
| 2 | 37 | Placebo | 30 | PD | Viable tumor |
|  | | | | | |
| 1 | 8 | 50 | 7 | PD | Viable tumor |
| 2 | 8 | 50 | 7 | PD | Viable tumor |
| 3 | 8 | 50 | 15 | SD | Viable tumor |
| 4 | 8 | 50 | 15 | PD | Viable tumor |
| 5 | 37 | 50 | 30 | PD | Viable tumor |

**Table S2. Summary of C/T790M bitransgenic mice treated with erlotinib.** PD – progressive disease; SD – stable disease. Responses were defined as described in the Methods.
