# Supplementary material for: Development of New Mouse Lung Tumor Models Expressing EGFR T790M Mutants Associated with Clinical Resistance to Kinase Inhibitors
Source: PLoS One. 2007 Aug 29;2(8):e810. doi: 10.1371/journal.pone.0000810 (PMC1950079; doi:10.1371/journal.pone.0000810)
Supplement: Table S3 — Summary of C/L858R+T790M bitransgenic mice (line 51) treated with 17-AAG. (0.09 MB DOC) [file pone.0000810.s003.doc]

| **Study no.** | **17-AAG dose (mg/kg/d)** | **Duration of treatment** | **Response by MRI** | **Status of EGFR by immunoblotting** | **Histology after 17-AAG treatment** |
| --- | --- | --- | --- | --- | --- |
| 1 | Placebo | 6h | nd | EGFR present | Not available |
| 2 | Placebo | 6h | nd | EGFR present | Viable tumor |
| 3 | Placebo | 3d | PD | nd | Tumor removed* |
| 4 | Placebo | 3d | PD | nd | Viable tumor |
| 5 | Placebo | 3d | PD | nd | Viable tumor |
|  | | | | | |
| 1 | 75 | 6h | nd | EGFR degraded | Not available |
| 2 | 75 | 6h | nd | EGFR degraded | Minimal response** |
| 3 | 75 | 6h | nd | EGFR degraded | Area of tumor necrosis |
| 4 | 75 | 3d | PR | nd | Area of tumor necrosis |
| 5 | 75 | 3d | PR | nd | Tumor removed |
| 6 | 75 | 3d | PR | nd | Tumor removed |
| 7 | 75 | 3d | PR | nd | Minimal response |
| 8 | 75 | 3d | PR | nd | Area of tumor necrosis |
| 9 | 75 | 3d | SD | nd | Area of tumor necrosis |
| 10 | 75 | 2w | PR | nd | Minimal response |
| 11 | 75 | 2w | PD | nd | Minimal response |
| 12 | 75 | 2w | PR | nd | Tumor removed |
| 13 | 75 | 4w | SD | nd | Viable tumor |
| 14 | 75 | 4w | SD | nd | Minimal response |

**Table S3. Summary of C/L858R+T790M bitransgenic mice (line 51) treated with 17-AAG.** h – hour; d – day; w – week; nd – not determined; PR – partial response; SD – stable disease; PD – progressive disease. Responses were defined as described in the Methods. *Histology from these samples may not have been representative of actual tumor burden, because the portion of lung where the tumor was identified by MRI was excised and flash frozen for other potential studies, while the remaining portion of the lungs sent for histological processing turned out not to have tumor nodules. **Minimal response: majority of tumor nodules viable but with evidence of some treatment effect. Note that even lungs that displayed areas of tumor necrosis often still demonstrated large adjacent areas of viable tumor.
