# Supplementary figures and images for: Development of New Mouse Lung Tumor Models Expressing EGFR T790M Mutants Associated with Clinical Resistance to Kinase Inhibitors
Source: PLoS One. 2007 Aug 29;2(8):e810. doi: 10.1371/journal.pone.0000810 (PMC1950079; doi:10.1371/journal.pone.0000810)

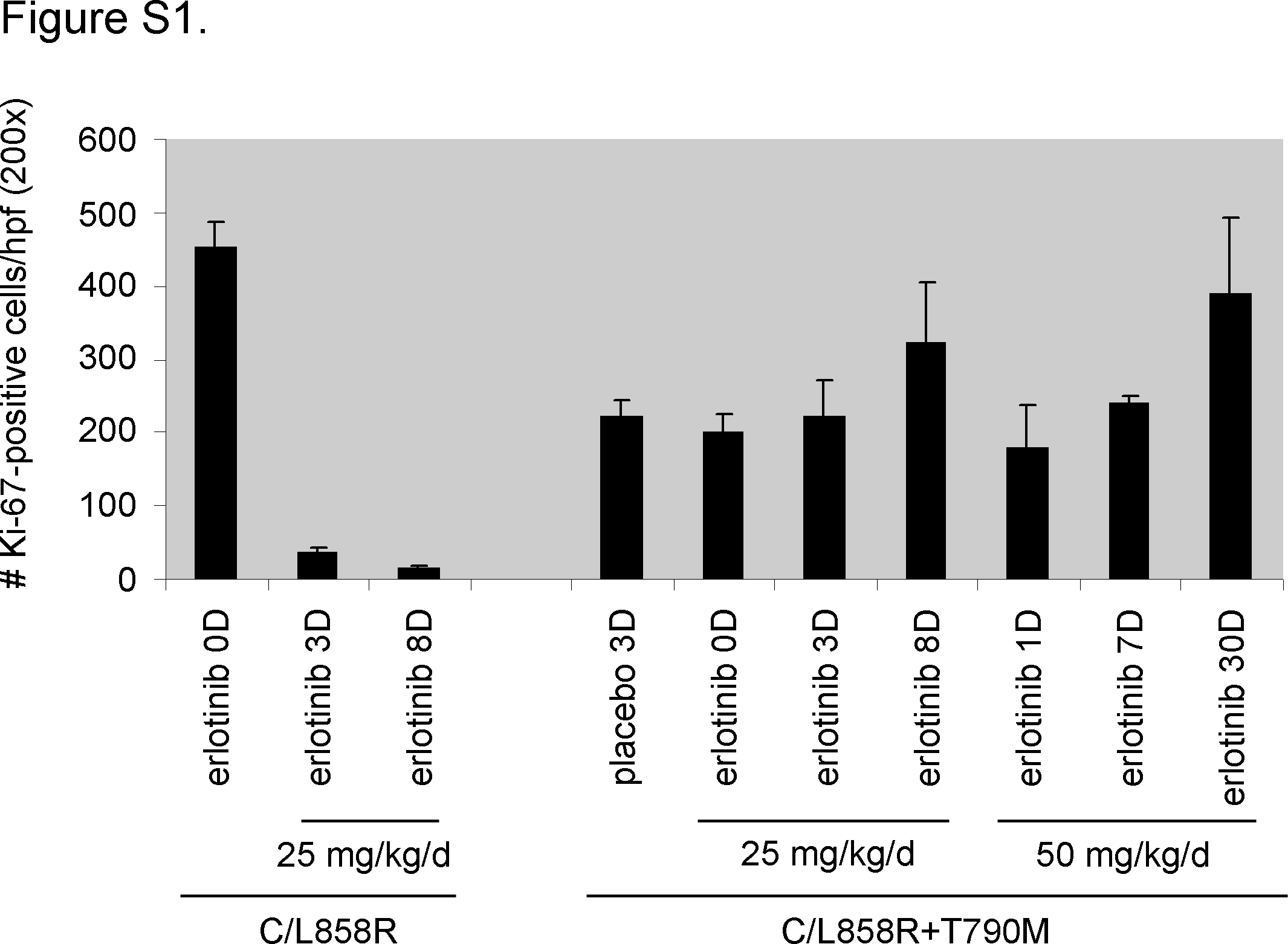

Supplement: Figure S1 — Status of cell proliferation in lung tumors from C/L858R and C/L858R+T790M animals treated with erlotinib. Histological sections derived from the lungs of bitransgenic animals were stained with antibodies to the proliferation marker, Ki-67. Ki-67-positive cells in lungs were then quantitated by determining the average (with standard deviations) number of positive cells counted in three separate high power fields (hpf) at a magnification of 200x. (3.34 MB TIF) [file pone.0000810.s004.tif]

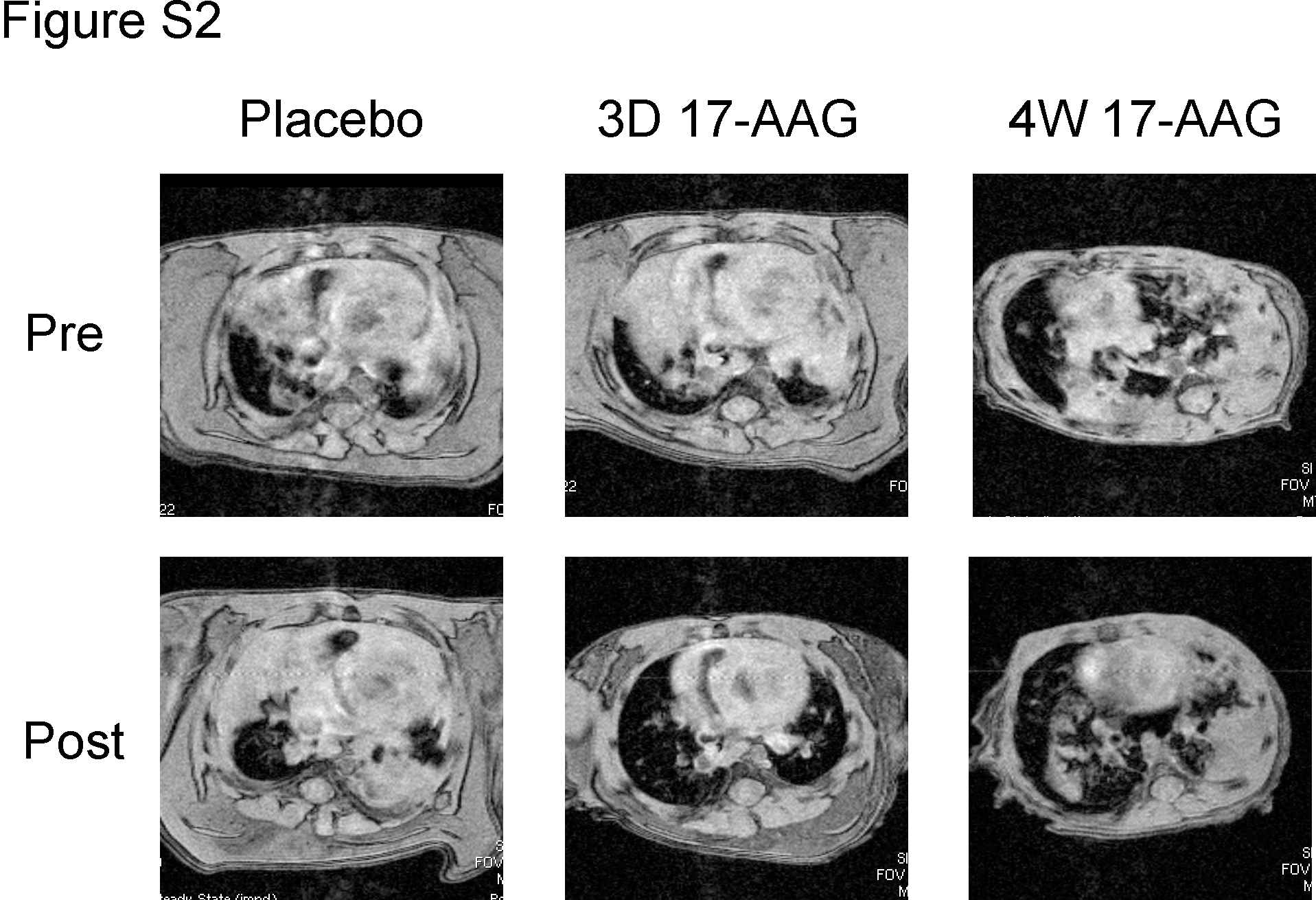

Supplement: Figure S2 — Representative MR images from bitransgenic mice treated with 17-AAG. Serial pre- and post-treatment images are shown. D-days; W-weeks. (7.55 MB TIF) [file pone.0000810.s005.tif]
